# Coexistence of YWHAZ amplification predicts better prognosis in muscle-invasive bladder cancer with CDKN2A or TP53 loss

**DOI:** 10.18632/oncotarget.9158

**Published:** 2016-05-04

**Authors:** Shenghua Liu, Yishuo Wu, Tian Yang, Chenchen Feng, Haowen Jiang

**Affiliations:** ^1^ Fudan Institute of Urology, Huashan Hospital, Fudan University, Shanghai 200040, China; ^2^ Department of Urology, Huashan Hospital, Fudan University, Shanghai 200040, China

**Keywords:** YWHAZ, copy number alteration, amplification, prognosis, bladder cancer

## Abstract

The amplification of YWHAZ was commonly seen in bladder cancer. We explore the biological significance of YWHAZ amplification on bladder cancer, and the correlation with important other molecular events. The Cancer Genome Atlas (TCGA) database was exploited to study the impact of YWHAZ amplification on either CDKN2A or TP53 mutations. The Database for Annotation, Visualization and Integrated Discovery (DAVID) was also exploited to clustering of enriched genes in the cBioPortal Enrichment tests. There were 127 cases with available mutation and CNV data in the corresponding TCGA bladder cancer dataset, 20% of them had YWHAZ alteration. Patients with both YWHAZ amplification and CDKN2A loss demonstrated significantly better overall survival (OS) compared with CDKN2A loss alone. Patients with both YWHAZ amplification and TP53 mutation demonstrated significantly better overall survival (OS) and disease-free survival (DFS) compared with TP53 mutation alone. The amplification of YWHAZ, along with alteration of CDKN2A or TP53, predict better survival in bladder cancers that only had CDKN2A or TP53 alteration. The protective role of YWHAZ in bladder cancer deserve insightful further studies.

## INTRODUCTION

Urinary bladder cancer remains one of the most commonly diagnosed cancers in the world. Around 75% of bladder cancer were non-muscle invasive, which is characterized by a high risk of recurrence and progression while the remaining 25% of bladder cancers were muscle-invasive, which is characterize by a high risk of metastasis and poor prognosis [1]. However, treatment progress of bladder cancer has been stagnant for the past decades [2].

One strategy to improve the care of bladder cancer patients is to explore the molecular pathogenesis and novel tumor markers to predict prognosis. Genetic alteration has been known to contribute to the occurrence and development of bladder cancer [3]. By integrated analysis of genomic event, we could define some subgroups of cancer type and found correlations between clinical information, which could provide robust prognosis markers.

Recent report using whole-genome sequencing techniques have identified varieties of focal copy number alterations of bladder cancer, including YWHAZ, located at 8q22, a gene involved in a wide range of biological processes [4]. The cocomitment gain of YWHAZ was found to be associated with some common molecular events, including TP53 mutation [5]. Up till now, the link between YWHAZ and biological characteristics of bladder cancer remains poorly understood. In our study, we reported using the online analytical tools of The Cancer Genome Atlas (TCGA) database, to explore the biological significance of YWHAZ amplification on bladder cancer, and the correlation with important other molecular events.

## RESULTS

**Figure 1:**
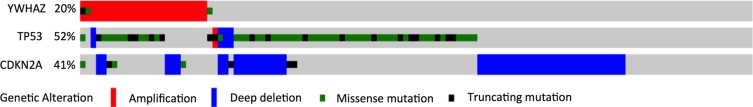
Gene alteration of YWHAZ, TP53 and CDKN2A in bladder cancer patients

**Figure 2:**
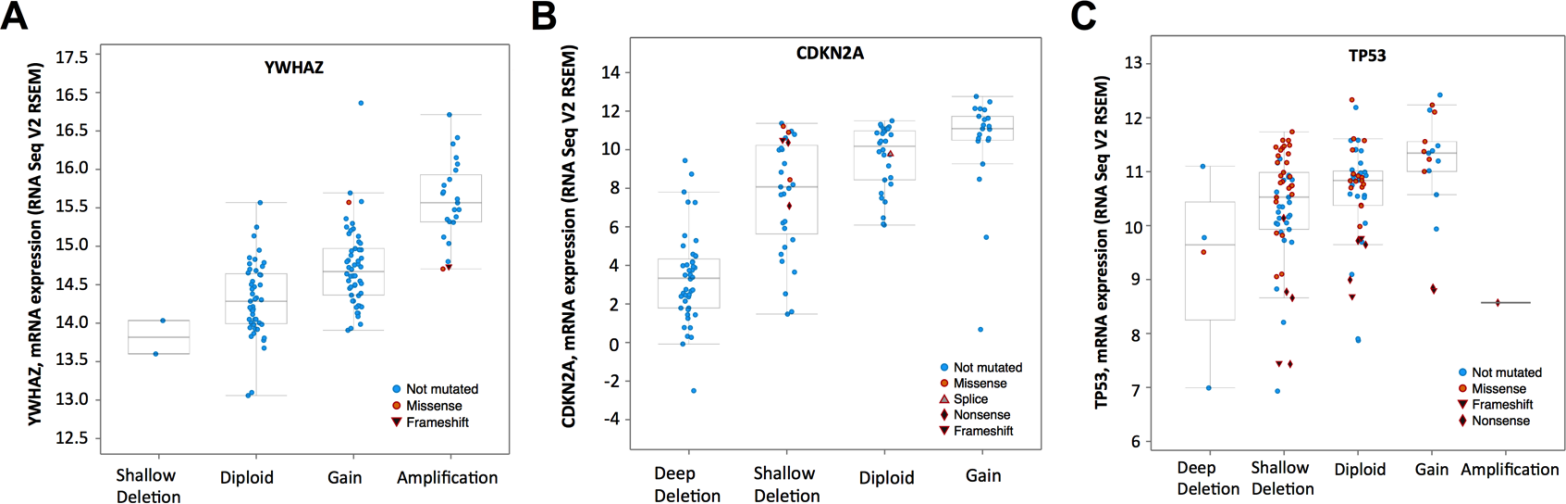
mRNA level was consistent with gene change Functional plotting of the corresponding mRNA level in relation to genetic status of (**A**) YWHAZ, (**B**) CDKN2A and (**C**) TP53.

There were 127 cases with available mutation and CNV data in the corresponding TCGA bladder cancer dataset. Amplification/mutation of YWHAZ was found in ~20% of cases (Figure 1). Amplification was the predominant type of alteration for YWHAZ. Deep deletion/mutation of CDKN2A was found in ~41% of cases, whilst Deep deletion/mutation of TP53 was found in ~52% of cases (Figure 1). There were 9 patients (7%) with YWHAZ and CDKN2A alterations, and 16 patients (12.5%) with both YWHAZ and TP53 alterations (Table 1 and Table 2). Functional plotting of the corresponding mRNA level in relation to genetic status of YWHAZ, TP53, and CDKN2A revealed that amplification of YWHAZ was associated with increased mRNA expression (Figure 2A); deletion of CDKN2A was associated with decreased mRNA expression (Figure 2B); mutation of TP53 was not obviously associated with mRNA expression but loss of copy number of was associated with lowered mRNA expression (Figure 2C).

**Table 1:** The characteristics of patients with TP53 alteration (+: altered; −: unaltered)

		YWHAZ (−) TP53 (+)	YWHAZ (+) TP53 (+)	*P* Value
Age (Mean ± SD)		67.69 ± 9.12	66.33 ± 11.11	0.823
Gender (*n*%)	Male	37 (56.1)	13 (19.7)	0.556
	Female	13 (19.7)	3 (4.5)	
T (*n*%)	2	14 (21.2)	4 (6.1)	0.925
	3	22 (33.3)	8 (12.1)	
	4	7 (10.6)	2 (3.0)	
	Tx	7 (10.6)	2 (3.0)	
N (*n*%)	0	28 (42.4)	6 (9.1)	0.215
	1	4 (6.1)	3 (4.5)	
	2	9 (13.6)	5 (7.6)	
	3	4 (6.1)	0 (0)	
	Nx	5 (7.6)	2 (3.0)	
M (*n*%)	0	26 (39.3)	10 (15.1)	0.385
	1	2 (3.0)	0 (0)	
	Mx	22 (33.3)	6 (9.1)	
Living Status (*n*%)	Living	21 (31.8)	10 (15.1)	0.153
	Decreased	29 (43.9)	6 (9.1)	

**Table 2:** The characteristics of patients with CDKN2A alteration (+: altered; −: unaltered)

		YWHAZ (−) CDKN2A (+)	YWHAZ (+) CDKN2A (+)	*P* Value
Age (Mean ± SD)		68.07 ± 9.50	68.33 ± 8.90	0.934
Gender (*n*%)	Male	32 (61.5)	7 (13.5)	0.802
	Female	11 (21.2)	2 (3.8)	
T (*n*%)	2	11 (21.2)	1 (1.9)	0.404
	3	22 (42.3)	5 (9.6)	
	4	6 (11.5)	0 (0)	
	Tx	4 (7.7)	3 (5.8)	
N (*n*%)	0	26 (50.0)	5 (9.6)	0.618
	1	4 (7.7)	1 (1.9)	
	2	8 (15.4)	0 (0)	
	3	1 (1.9)	0 (0)	
	Nx	4 (7.7)	3 (5.8)	
M (*n*%)	0	26 (50.0)	3 (5.8)	0.252
	1	2 (3.8)	1 (1.9)	
	Mx	15 (28.8)	5 (9.6)	
Living Status (*n*%)	Living	19 (36.5)	6 (11.5)	0.22
	Decrease	24 (46.2)	3 (5.8)	

**Figure 3:**
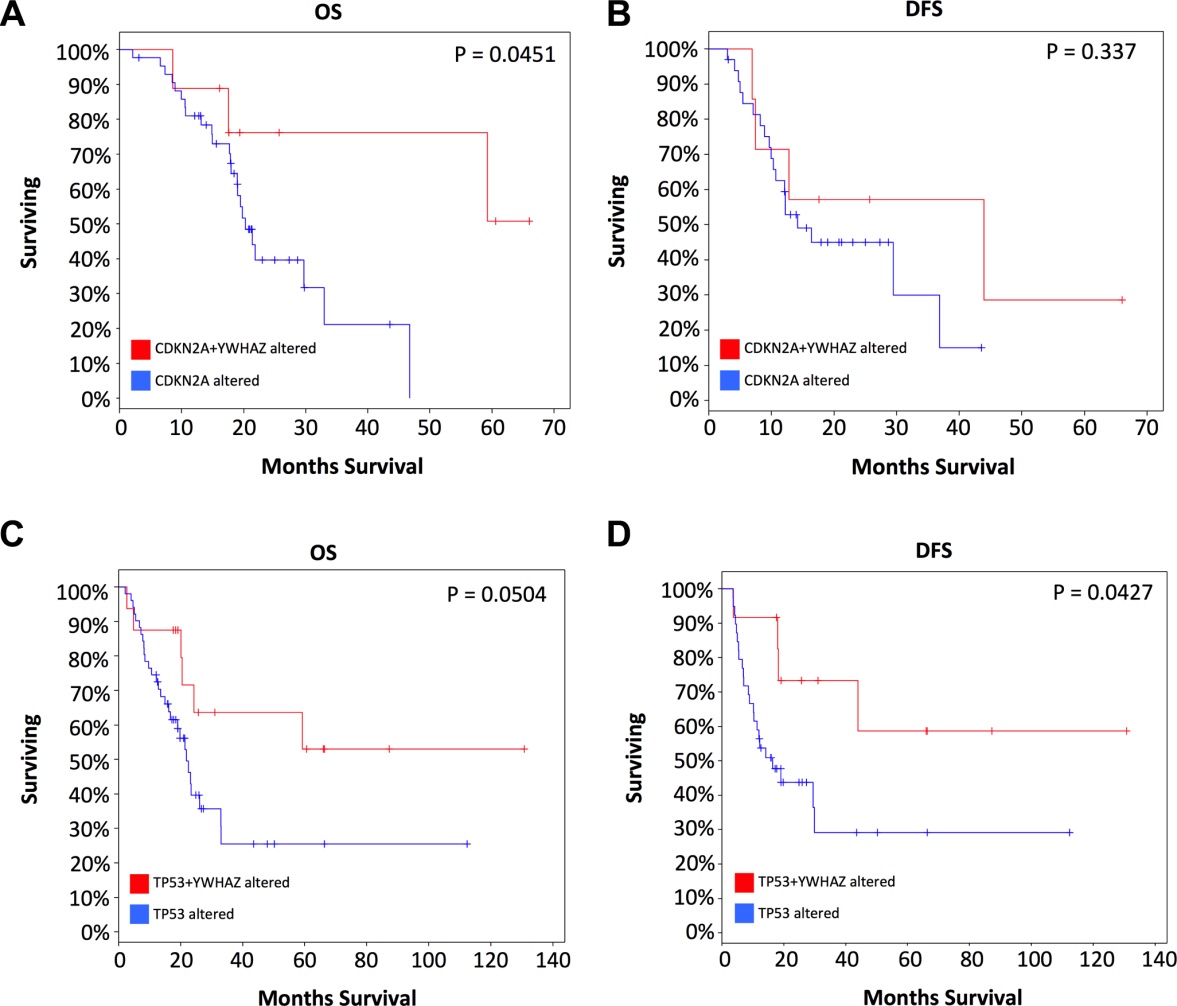
Coexistence of YWHAZ amplification contribute to better prognosis with CDKN2A or TP53 loss

**Figure 4:**
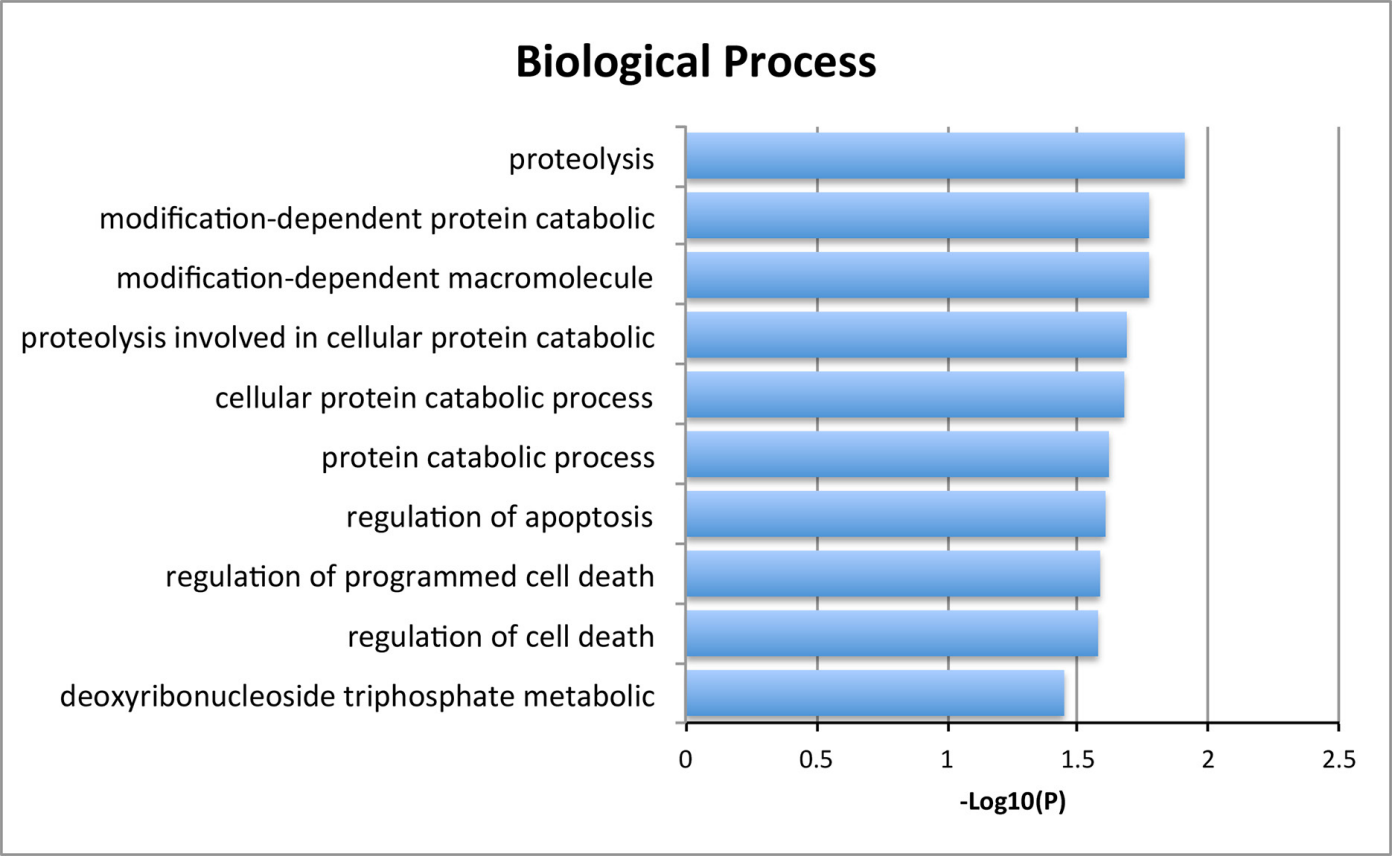
Biological process of the significantly enriched genes between both YWHAZ and TP53 alteration and single TP53 alteration

**Figure 5:**
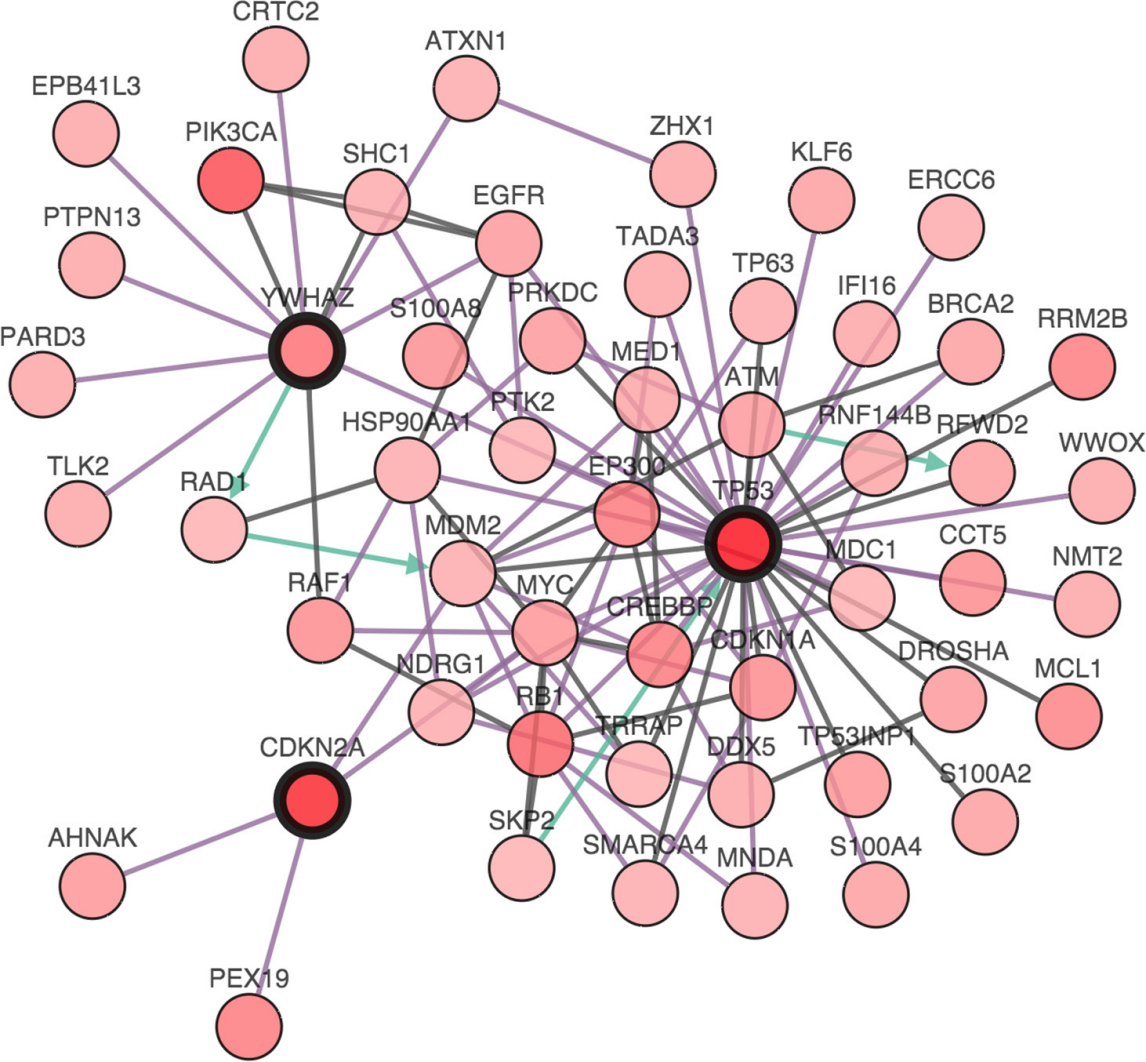
The interactions between TP53, CDKN2A, and YWHAZ alteration

We then studied cases with alterations in either gene, namely CDKN2A, or TP53 and grouped cases according to co-occurrence of YWHAZ amplification. Demographic and clinicopathological parameters were summarized in Tables 1 and 2, and age, gender, T stage, nodal involvement, metastasis, or living status was not significantly different between TP53 mutated or CDKN2A deleted patients with or without YWHAZ amplification (Table 1 and Table 2). Patients with both YWHAZ amplification and CDKN2A loss demonstrated significantly better overall survival (OS) compared with CDKN2A loss alone (Figure 3A). Nonetheless, the disease-free survival (DFS) was not significantly different (Figure 3B). Patients with both YWHAZ amplification and TP53 mutation demonstrated significantly better overall survival (OS) and disease-free survival (DFS) compared with TP53 mutation alone (Figure 3C–3D). For CDKN2A, cases with both alterations versus single alteration did not demonstrate significant differences in enrichments at mRNA, protein, CNV, or mutation level (data not shown). For TP53, cases with both alterations versus single alteration demonstrated a series of significantly enriched genes at mRNA expression level. When those genes were input into DAVID, we noticed that proteolysis was the top enriched biological process between patients with both TP53 mutation and YWHAZ amplification, and patients with solely TP53 mutation (Figure 4). We then tried to analysis the interactions between TP53, CDKN2A, and YWHAZ alteration via computation, yet the strength of direct interaction between the nodes was solely moderate (Figure 5).

We summarized the CNA and expression status of YWHAZ based on the study of Cancer Cell Line Encyclopedia. A total of 54 breast cancer, 7 prostate cancer and 24 urinary tract cancer cell lines were included. The profile of YWHAZ change and its mRNA expression status in this three cancer types was showed in Table 3. Details in different cell lines was showed in Supplementary Table 1.

**Table 3:** The profile of YWHAZ gene and expression status in breast, prostate and urinary tract cancer cell lines

cancer type	YWHAZ status	Cell lines count
breast	amplification	9
	mRNA upregulation	5
	mRNA downregulation	1
	unchanged	45
prostate	amplification	1
	unchanged	6
Urinary tract	mRNA upregulation	3
	unchanged	21

## DISCUSSION

This study revealed that the amplification of YWHAZ, along with alteration of CDKN2A or TP53, predict better survival in bladder cancers that only had CDKN2A or TP53 alteration. The finding implicated that YWHAZ might play protective roles in bladder cancer. This was interesting because in previous studies, the overexpression of YWHAZ, has been implicated in the initiation and progression of multiple cancers [6]. In prostate cancer, 14-3-3ζ was upregulated by androgen and contributes to cell proliferation and resistance to etoposide-induced apoptosis in LNCaP cells. The higher expression is associated with malignancy and lymph node metastasis [7]. In breast cancers, 14-3-3ζ overexpression occurred in nearly half of breast tumors and was determined to be an independent prognostic factor for reduced disease-free survival. By using stable transfection or siRNA technique, increased 14-3-3ζ expression was found to enhanced cell growth and inhibited apoptosis, whereas downregulation of 14-3-3ζ reduced growth and sensitized cells apoptosis in breast cell lines [8].

TP53, also known as tumor protein 53, is a well-known tumor suppressor gene. The mutation or deletion of TP53 gene is commonly seen in bladder cancer patients [9]. In our cohort, TP53 mutation or deletion is seen in 52% of the patients. CDKN2A, also known as cyclin-dependent kinase inhibitor 2A, codes for two proteins, including p16 an p14arf, both act as tumor suppressors by regulating the cell cycle. The genetic alteration of CDKN2A is most common clinically relevant in the advanced bladder cancer [10]. Both TP53 and CDKN2A alteration contributed to the unfavorable clinical and survival outcomes [11].

In muscle invasive bladder tumor, gain on 8q was paralleled with TP53 mutation. Here using TCGA database we found this association positively contributed to the overall survival. Also the survival benefits were observed in the combination of YWHAZ and CDKN2A alteration. As both TP53 and CDKN2A were cell cycle checkpoint genes, we assume YWHAZ function in the cell cycle related pathway. 14-3-3 zeta regulates cell cycle progression through various ligands and processes. Previous study reported that 14-3-3 zeta controls cellular senescence by complexing with BIS to chaperone protein folding of STAT3 and activate the signaling pathway [12]. Also, 14-3-3 zeta can negatively regulate the G2-M phase checkpoint by binding and sequestering the cyclin-dependent kinases to the cytoplasm, thus inhibiting their activity [13]. Another study reported 14-3-3 zeta binding to Cdc25B and inhibits its interaction with CyclinB/Cdk1 [14]. Cdc25 dual-specificity phosphatases are essential regulators that activate cyclin-dependent kinases(CDKs) at critical stages of the cell cycle, acting both in S phase and G2/M in mammalian cells [15]. Thus, developing selective inhibitors for Cdc25 family proteins could provided novel therapeutic strategies for cancer therapy [16–18]. This might account for how 14-3-3 zeta exerts its protective function. As a tumor suppressor, CDKN2A is involved in the p53 pathway, arrest growth by holding the cell cycle at G1/S checkpoint on DNA damage recognition. When this mechanism fails, 14-3-3 zeta could inhibit Cdc25B and arrest the cell cycle at G2/M phase. All these speculations however still warrant insightful studies both *in vivo* and *in vitro*.

## MATERIALS AND METHODS

An *in silico* reproduction using TCGA dataset was performed in the current study, as previously reported [19–21]. The TCGA bladder cancer (Provisional) dataset was chosen on the cBioPortal online platform [22, 23]. Cases with CDKN2A or TP53 mutations were respectively chosen, amongst which concurrent YWHAZ amplification was queried using the OncoPrint function. The Plots function illustrated the correlation of CNV/mutation versus mRNA expression. The Enrichment function explored alterations including mutations, copy number alterations, mRNA expression changes, and protein expression changes that were enriched in either altered or unaltered queried samples. The Survival illustrated Kaplan-Meier curves for overall survival and cancer-free survival. The Network illustrated the interactions between queried genes and in our cohort. All statistical analyses were performed automatically by the cBioPortal platform and the *P* value of < .05 and *Q* value of < .05 were accepted as statistically significant, respectively.

To explore the copy number alteration and mRNA expression status of YWHAZ on different cancer type, the Cancer Cell Line Encyclopedia (CCLE) datasets was chosen on cBioPortal online platform. Cell lines of breast cancer, prostate cancer, and urinary tract cancer was selected, amongst which YWHAZ was queried using OncoPrint function.

The Database for Annotation, Visualization and Integrated Discovery (DAVID) v6.7 was used for clustering of enriched genes passing both *p* (< .05) and *q* (< .05) values in the cBioPortal Enrichment tests. Clinicopathological parameters of the patients within TCGA cohort were retrieved and grouped according to alterations in single or double query genes and were compared using the Fisher's exact test. The *P* value of < .05 was accepted as statistically significant.

## SUPPLEMENTARY MATERIALS TABLE




