# Supplementary material for: Coexistence of YWHAZ amplification predicts better prognosis in muscle-invasive bladder cancer with CDKN2A or TP53 loss
Source: Oncotarget. 2016 May 4;7(23):34752–8. doi: 10.18632/oncotarget.9158 (PMC5085186; doi:10.18632/oncotarget.9158)
Supplement: Supplementary file 1 [file oncotarget-07-34752-s001.pdf]

Coexistence of YWHAZ amplification predicts better prognosis in muscle-invasive bladder cancer with CDKN2A or TP53 loss

Supplementary Materials

Supplementary Table S1: YWHAZ gene and expression status on different cell lines. See Supplementary_Table_S1
