# Supplementary material for: Coexistence of YWHAZ amplification predicts better prognosis in muscle-invasive bladder cancer with CDKN2A or TP53 loss
Source: Oncotarget. 2016 May 4;7(23):34752–8. doi: 10.18632/oncotarget.9158 (PMC5085186; doi:10.18632/oncotarget.9158)
Supplement: Supplementary file 2 [file oncotarget-07-34752-s002.docx]

**Supplementary Table S1: YWHAZ gene and expression status on different cell lines**

| **cell line** | **Tumor Type** | **amplification** | **mRNA upregulation** | **mRNA downregulation** |
| --- | --- | --- | --- | --- |
| Hs_274_T | breast |  |  |  |
| MDA-MB-157 | breast |  |  |  |
| ZR-75-30 | breast | yes | yes |  |
| AU565 | breast |  |  |  |
| JIMT-1 | breast |  |  |  |
| HCC1500 | breast |  |  |  |
| SK-BR-3 | breast |  |  |  |
| KPL-1 | breast | yes |  |  |
| HCC2157 | breast |  |  |  |
| HCC202 | breast |  |  |  |
| HCC1569 | breast |  |  |  |
| YMB-1 | breast |  |  |  |
| HCC1187 | breast |  |  |  |
| HCC1599 | breast |  |  |  |
| DU4475 | breast |  |  |  |
| MDA-MB-415 | breast |  |  |  |
| EFM-19 | breast |  |  |  |
| HCC1395 | breast |  |  |  |
| HCC1954 | breast | yes | yes |  |
| HCC1143 | breast |  |  |  |
| HCC1937 | breast |  |  |  |
| BT-483 | breast |  | yes |  |
| BT-474 | breast |  |  |  |
| HCC70 | breast |  |  |  |
| MDA-MB-453 | breast | yes |  |  |
| HCC38 | breast |  |  |  |
| MDA-MB-436 | breast |  |  | yes |
| MDA-MB-468 | breast |  |  |  |
| CAL-51 | breast |  |  |  |
| BT-549 | breast |  |  |  |
| T-47D | breast |  |  |  |
| MDA-MB-231 | breast |  |  |  |
| UACC-812 | breast |  |  |  |
| Hs_578T | breast | yes |  |  |
| Hs_739_T | breast |  |  |  |
| CAL-85-1 | breast |  |  |  |
| CAL-148 | breast |  |  |  |
| BT-20 | breast |  |  |  |
| MDA-MB-134-VI | breast |  |  |  |
| UACC-893 | breast | yes |  |  |
| CAL-120 | breast |  |  |  |
| HCC2218 | breast |  |  |  |
| EFM-192A | breast |  |  |  |
| Hs_742_T | breast |  |  |  |
| Hs_343_T | breast |  |  |  |
| HCC1428 | breast | yes | yes |  |
| ZR-75-1 | breast |  |  |  |
| HCC1806 | breast |  |  |  |
| HCC1419 | breast | yes | yes |  |
| MCF7 | breast | yes |  |  |
| HDQ-P1 | breast |  |  |  |
| MDA-MB-175-VII | breast |  |  |  |
| Hs_281_T | breast |  |  |  |
| Hs_606_T | breast |  |  |  |
| MDA-MB-361 | breast |  |  |  |
| CAMA-1 | breast |  |  |  |
| NCI-H660 | prostate |  |  |  |
| DU_145 | prostate |  |  |  |
| LNCaP_clone_FGC | prostate |  |  |  |
| 22Rv1 | prostate |  |  |  |
| PC-3 | prostate | yes |  |  |
| VCaP | prostate |  |  |  |
| MDA_PCa_2b | prostate |  |  |  |
| 5637 | Urinary tract |  |  |  |
| JMSU-1 | Urinary tract |  |  |  |
| UM-UC-1 | Urinary tract |  |  |  |
| UM-UC-3 | Urinary tract |  |  |  |
| TCCSUP | Urinary tract |  | yes |  |
| KU-19-19 | Urinary tract |  |  |  |
| SW-1710 | Urinary tract |  |  |  |
| CAL-29 | Urinary tract |  |  |  |
| 647-V | Urinary tract |  |  |  |
| HT-1376 | Urinary tract |  |  |  |
| 639-V | Urinary tract |  |  |  |
| HT-1197 | Urinary tract |  |  |  |
| KMBC-2 | Urinary tract |  |  |  |
| BFTC-905 | Urinary tract |  |  |  |
| RT-112 | Urinary tract |  |  |  |
| RT112_84 | Urinary tract |  |  |  |
| Hs_172_T | Urinary tract |  |  |  |
| BC-3C | Urinary tract |  | yes |  |
| VM-CUB1 | Urinary tract |  | yes |  |
| SW_780 | Urinary tract |  |  |  |
| SCaBER | Urinary tract |  |  |  |
| T24 | Urinary tract |  |  |  |
| J82 | Urinary tract |  |  |  |
| RT4 | Urinary tract |  |  |  |
